# The damage and reconstruction of the Kumamoto earthquake: an analysis on the impact of changes in expenditures with multi-regional input–output table for Kumamoto Prefecture

**DOI:** 10.1186/s40008-022-00276-6

**Published:** 2022-10-18

**Authors:** Kenta Takeda, Kazuo Inaba

**Affiliations:** 1 The General Incorporated Association Institute for Policy and Sciences, 1-3 Asahigaoka, Fujieda-shi, Shizuoka-ken 426-0081 Japan; 2 Faculty of Economics, Ritsumeikan University, 1-1-1 Noji-higashi, Kusatsu-shi, Shiga-ken 525-8577 Japan

**Keywords:** Regional input–output table, Multi–regional input–output analysis, 2016 Kumamoto earthquake, Kumamoto Prefecture, Economic ripple effects, R11, R15

## Abstract

The Kumamoto earthquake which occurred in April 2016 measured twice the maximum seismic intensity of 7, causing serious damage to the Kumamoto Prefecture. This study mainly focuses on the demand side of expenditures, estimating the monthly expenditures for 1 year before and after the earthquake. Then, using the multi-regional input–output table for Kumamoto Prefecture, we analyzes the ripple effects by region of the changes in monthly expenditures due to the earthquake. Expenditures in the prefecture in fiscal year 2016 by month decreased by a cumulative total of 592 billion yen because of the earthquake, which generated a value-added loss of 348 billion yen. On the other hand, expenditures increased by a cumulative total of 648 billion yen caused by reconstruction demand, inducing 375 billion yen in value-added gains. Thus, net increase of the value-added of 27 billion yen occupied 10.9% of net increase of the gross prefectural domestic product between fiscal years 2015–2016. The fluctuation of expenditures, induced production, and induced value-added caused by the earthquake is huge. Although the damage to the prefectural economy was severe, reconstruction demand exceeded it, resulting in a quick recovery. However, at the same time, there was a confirmed delay in restoration in industries that were almost unrelated to reconstruction and in regions with a heavy concentration of damage.

## Introduction

The destructive earthquake that occurred in April 2016 with twice the maximum seismic intensity of 7 in the Kumamoto region caused enormous damage to the entire prefecture. In addition to human casualties and housing damage, the damaging of products and capital equipment in primary and secondary industries caused outrage and a stagnation of production activities. The subsequent destruction of the supply chain spread the negative impact across the country.

The stagnation of production activities decreased corporate surplus and workers’ income and resulted in the reduction of consumer demand and capital formation. Furthermore, anxiety for the future and the reputational damage of the local people due to the earthquakes caused a decline in consumerism and tourism. On the other hand, the active support by the central and local government for the restoration of the infrastructure and the reconstruction of business and personal activities for the local people had an impact on raising consumer demand and capital formation. Thus, the serious damages from the earthquake may have been compensated for by the public expenditure and the people’s efforts to overcome the difficulties they faced. The physical and economic impacts of the earthquake may differ by region.

**Fig. 1:**
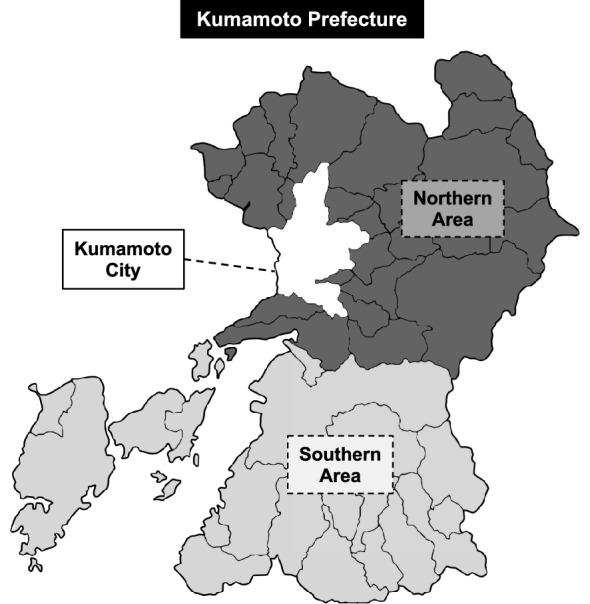
Area classification of Kumamoto Prefecture

The purpose of this study is to use the multi-regional input–output (MRIO) table to investigate the economic impacts of the earthquake in the regions of the Kumamoto Prefecture and the degree of recovery made by the restoration and reconstruction program. While an input–output analysis on the supply constraint model reflects an estimation of loss due to disasters, such as the Kumamoto earthquake, this study focuses on the demand side, which involves the change of expenditures in the prefecture by “damage” and “reconstruction.” We elucidate these effects on each region in the prefecture. Two databases, “Regional Domestic Expenditure Index” (RDEI) and “Prefectural Accounts,” enable this study to estimate the expenses of “damage” and “reconstruction” by month and the measure change of spending that annually based data does not grasp. We also estimate their positive and negative economic ripple effects using the MRIO table, which consists of three regions in the Kumamoto Prefecture (Kumamoto City, Northern, and Southern prefectures) as well as other parts of Japan. Figure 1 shows the prefecture and its area classification subject to this analysis.^1^

The structure of this paper is as follows: the next section explains the damage and reconstruction of the earthquake for background and reviews previous literature. The third section describes the methodology for the estimation and analysis of changes in expenditures and those ripple effects. The fourth section discusses the estimation result. The last section is the conclusion.

## Background

### Overview of the Kumamoto Earthquake

On April 14th, 2016, a magnitude 6.5 earthquake that measured a seismic intensity of 7 struck the Kumamoto region. Furthermore, 2 days after, a magnitude 7.3 earthquake that measured an intensity of 7 struck the region again. 4484 aftershocks over an intensity of 1 were recorded through March 2018.^2^ These disastrous earthquakes resulted in many casualties centered in the Kumamoto Prefecture, caused in part by houses collapsing, liquefaction, and sediment disasters. There are 3009 confirmed casualties in Kumamoto Prefecture (dead: 273, injured: 2736) including related damage. Approximately 198,000 homes were damaged (completely destroyed: 8642, half: 34,389, partially: 155,227).^3^

In addition, production facilities, equipment, and stores related to agriculture, forestry, fisheries, commerce and industry, and social infrastructure such as water, sewage, electricity, gas facilities and roads, as well as cultural assets were also severely damaged. The prefectural office and the Cabinet office estimated the capital stock damage at 3.8 trillion yen, which accounted for 11% of the prefecture’s total capital stock of 34 trillion yen. The Cabinet office evaluated approximately 81–113 billion yen as the amount of flow damage during the month after the earthquake.^4^ Lodging cancellations also reached 330,000 nights.^5^

### Damage and Reconstruction

Whereas torrential rain, typhoons, and COVID-19 struck the region after the earthquake, reconstruction is progressing steadily. The maximum number of evacuees recorded was 184,000 just after the earthquake. 90% of evacuees were able to leave the shelters 1 month later, and all shelters were eliminated by November 2016.^6^ 48,000 people still had to move into temporary housing because of the enormous damage to housing. Thereafter, 70% of occupants were able to move into new homes within 2 years, but 95 people are still forced to live in temporary housing.^7^

First, the damage in the regions targeted for analysis is reviewed. The earthquake, with its epicenter in Kamimashiki County in the northern area of the prefecture just southeast of Kumamoto City in Fig. 1, caused extensive damage mainly from the city to the northern area. Table 1 shows human casualties and building damages by region in the prefecture. Kumamoto City, which has a high population density and many apartment complexes, had the most injured. In addition, number of partially destroyed of housing was highest due to close to the epicenter and frequent liquefaction. Northern prefecture, which located in the epicenter and recorded twice an intensity of 7, has most deaths and number of completely and half destroyed housing units and other building damage. The strong tremors and resulting landslides also caused extensive damage to roads, bridges, and other public works facilities, and to municipal government buildings. In Southern prefecture, which is relatively far from the epicenter, the damage was less damage to both people and buildings. However, there is relatively more damage to public and other facilities, including some municipal government buildings that had become unusable in the area near the epicenter.

**Table 1:** Maximum intensity and damage of human and building by region

	Kumamoto Prefecture				Regional ratio		
		Kumamoto City	Northern prefecture	Southern prefecture	Kumamoto City	Northern prefecture	Southern prefecture
Maximum intensity	7	6+	7	6−	–	–	–
Human damage (unit: people)	3009	1803	1164	42	59.9%	38.7%	1.4%
Death	273	88	177	8	32.2%	64.8%	2.9%
Injured	2736	1715	987	34	62.7%	36.1%	1.2%
Seriously	1186	772	402	12	65.1%	33.9%	1.0%
Slightly	1550	943	585	22	60.8%	37.7%	1.4%
Building damage	211,395	122,904	82,955	5536	58.1%	39.2%	2.6%
Housing	198,258	122,761	71,018	4479	61.9%	35.8%	2.3%
Completely destroyed	8,642	2456	6131	55	28.4%	70.9%	0.6%
Half	34,389	15,219	18,537	633	44.3%	53.9%	1.8%
Partially	155,227	105,086	46,350	3791	67.7%	29.9%	2.4%
Non-housing	13,137	143	11,937	1057	1.1%	90.9%	8.0%
Public	467	60	253	154	12.8%	54.2%	33.0%
Other	12,670	83	11,684	903	0.7%	92.2%	7.1%

“Information on the 2016 Kumamoto Earthquake,” Japan Meteorological Agency website. “Damage from the Kumamoto Earthquake [Report 326],” Kumamoto Prefecture

**Fig. 2:**
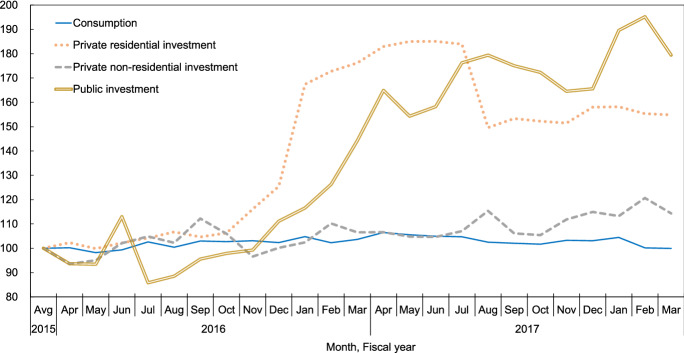
Regional Domestic Expenditure Index. Source: Based on the Regional Domestic Expenditures Index, the Cabinet Office**.** Indices are adjusted to average FY2015 = 100. The fixed capital formation is evaluated by actual volume. The public mechanical equipment data have a 3-month lag

Second, review the changes on the demand side after the earthquake based on some economic statistics. Figure 2 shows that starting in September 2016, the synthesis consumption index remained above the FY2015 average until March of FY2017. According to the Family Income and Expenditure Survey (FIES),^8^ increased spending on furniture and electrical appliances in April 2017 suggests temporary restoration demand. Then, after the reduction in spending, the project for the revitalization of production activity and rebuilding livelihood stimulated demand.

Regarding private residential investment, while the level was slightly below the FY2015 average in May 2016, it has been over the average since then, with rapid growth since November 2016. According to the Building Starts Statistics (BSS), the construction floor area is beginning to expand since October 2016. In addition, the number of housings starts above the FY2015 level has cumulatively totaled 9587 through 2018, more than the number of housing units completely destroyed by the earthquake.^9^

In May 2016, private non-residential investment recovered and exceeded the previous fiscal year’s average from June through September 2017. After being below the average in November, its level immediately recovered and has been high ever since. After the fluctuation between April and July 2016, public investment gradually increased, and since December 2016, it has remained well above the average of the previous fiscal year.

Emergency restoration demand rose immediately after the earthquake. Thereafter, although the reaction to the emergency spending and the shortage of construction-related manpower and equipment made demand decrease or slow down, full-fledged demand for reconstruction rose as production activities were revitalized by facility restoration projects, subsidies, and special loans.

**Fig. 3:**
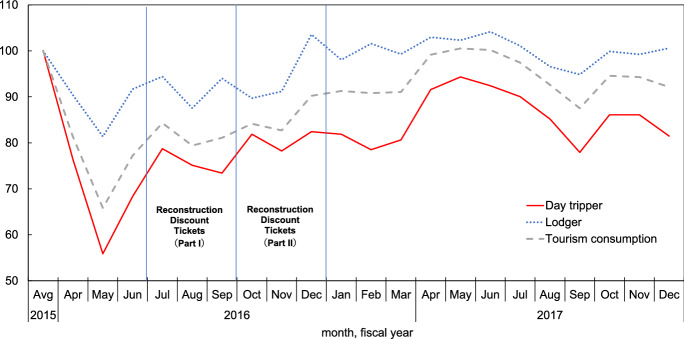
Index of tourists and tourism consumption. Source: Based on the Kumamoto Tourism Statistics, Kumamoto Prefecture. Average in FY2015 = 100. Seasonally adjusted. Tourism consumption is the number of tourists multiplied by per capita spending

Looking at the number of tourists in Fig. 3, both day trippers and lodgers dropped dramatically in April and May 2016 and rose incrementally after July 2016 thanks to “Kyushu Fukko Wari (Kyushu Reconstruction Discount Tickets)^10^”. At the end of 2016, the number of overnight visitors exceeded the average level for FY2015 and then remained steady. Nevertheless, the number of day trippers did not reach the FY2015 average within 2017.

Appendix Table 4 shows that while overall, the gross prefectural domestic production (GPDP, expenditure approach) increased as a whole, household final consumption expenditure, private non-residential investment, and inventory changes fell. The reduction in demand for clothing and footwear, housing, and entertainment is especially conspicuous. On the other hand, government demand grew markedly in both consumption and capital formation for restoration and reconstruction.

### Previous studies

There are two approaches to discuss the economic impacts of the earthquake: supply side and demand side.

The former approach focuses on the impacts of the disruption of the supply chain to evaluate production loss caused by the earthquake. Hasebe (2002) proposes a supply constraint model based on the assumption of an earthquake directly below the Tokyo area and estimated the production decline associated with the earthquake. The model focuses on the headquarters function, which is difficult to substitute, and quantitatively evaluates the impact of bottleneck supply constraints due to the cessation of the headquarters function, assuming a perfect non-substitutional Leontief production function. Shimoda and Fujikawa (2012) examine a supply constraint model based on the assumption of the 2011 Great East Japan Earthquake. They measured the impact of the production decline in the Tohoku region on other regions using four models: the Leontief model (backward linkage), the Ghosh model (forward linkage), the hybrid model (forward and backward linkage), and the bottleneck model (Hasebe 2002), and compared the results. The paper points out the usefulness and problems of each model. Regarding the Kumamoto earthquake, Kanzaki and Okamoto (2017) estimate backward linkage effects of production decline of agriculture, forestry, fisheries, and the manufacturing industry in the prefecture on economic activities of other parts of Japan using inter-prefectural input–output model. They identify differences in the regional spread of ripple effects by industry using a unique index called multi-reginal interaction reduction rate and also discuss financial measures for the earthquake, calling for the need for proactive financial measures not only for direct stock damage but also for indirect flow damage. Okiyama and Tokunaga (2018) calculate decline in production of agriculture, forestry, and fisheries in Kumamoto Prefecture which multiplies stock damage by production per capital stock, and measure forward linkage effects with Ghosh model based on inter-prefectural input–output table. Moreover, they assume that similar damage would have occurred in other prefecture, and by estimating and comparing the ripple effects, they clarify the differences in ripple patterns between metropolitan and non-metropolitan areas.

On the other hand, the latter approach focuses on amount of damage, economic ripple effects by restoration activities, and the process of reconstruction. Ashiya and Jinushi (1999) estimate the economic impact of construction investment involving reconstruction activities from the Great Hanshin–Awaji Earthquake. They compile the input–output table for quake-hit area when before and after the earthquake for analysis of structural change on the area. Using these tables and data of damage, they clarify the ripple effects based on economic structure after the hit. Nakano (2011) assesses the impact of the drop in production and final demand in the areas affected by the Great East Japan Earthquake on employment in Japan. In that paper, the rate of decrease in production is calculated based on the damage to buildings, thereby estimating the amount of decrease in production and demand. In addition, by assuming multiple cases based on the substitutability of goods and by estimating and the ripple effects, the paper shows that the higher the non-substitutability of goods, the worse the impact on employment. As for the Kumamoto earthquake, Cui (2016) estimates the impact of the decline in number of tourists on the economy of Kumamoto City. At first, he estimates decline in the number of tourists during the recovery process of Himeji Castle and assumes that the restoration process of Kumamoto Castle would follow the same transition as the case in Himeji Castle, where the rate of decrease in tourists and the recovery process during the restoration is estimated. He applies this method to the case in Kumamoto and estimates the negative ripple effects by calculating the amount of decrease in tourists and tourism consumption. Kato and Honjo (2016) discuss, from a branch plant perspective, the differences in the recovery status of local industries experiencing a great disaster for the first time and those of affiliates of major companies that have already experienced recovery from the earthquake. They evaluate the effects of the earthquake on the prefecture's economy in terms of a fall in production in the manufacturing industry and lodging cancellations in Kumamoto Prefecture. All of the demand side approach listed here are estimated as backward linkage effects using the Leontief model.

In recent years, the trend in input–output analysis of disasters has tended to emphasize the evaluation of production losses (e.g., supply constraints). In the case of the Kumamoto earthquake, demand-side analysis is limited to tourism and some manufacturing industries, while analysis of household consumption, investment, and government spending is insufficient. The supply constraint model measures the impact of supply chain disruptions and tends to focus on effects outside the affected areas, with little reference to spillover effects within Kumamoto Prefecture.^11^ In addition, all approaches only analyze the negative ripple effects from the decrease in production and tourists, and do not mention the positive effects due to the expansion of reconstruction demand. Furthermore, while damage from the earthquake varies by region, most of the analyses do not clarify the damage and its effects by and among regions within the prefecture. Although, since these analyses are conducted at a relatively early stage after the occurrence of the earthquake with limited data availability, their methodologies and value as a preliminary report are appreciated.

Based on the previous studies, this study focuses on demand side and analyze impacts of change in expenditure by region and month due to damage and reconstruction demand caused the Kumamoto earthquake using multi-regional input-out table for Kumamoto Prefecture, which we estimate independently. This study follows these demand side approach and estimates backward linkage effects by multi-regional Leontief model.

## Methods and analytical framework

### Compilation of multi-regional input output table for Kumamoto

This study uses the MRIO table for Kumamoto Prefecture in 2015, which consists of 105 industrial sectors and 4 regions: Kumamoto Prefecture (Kumamoto city, Northern region, and Southern region), and the other parts of Japan. Using a non-survey method, the intra-regional input–output tables each region are compiled based on the input–output (IO) tables for the prefecture and Japan, and other statistics. Inter-regional transactions were estimated by referring Maekawa (2012)'s method. The method divides transactions of inter-reginal IO table which consist of two regions between inside and outside a prefecture into targeted areas and the other parts by production and demand share. This method is extended to divide transactions in the prefecture into Kumamoto City and the other area, and then divide transactions in the other area into northern and southern areas. These compiled tables are rearranged to Chenery–Moses type model. Moreover, they are applied to the Isard type.^12^

### Estimation of expenditure change by damages from the earthquake and the reconstruction process

Monthly base expenditure is used to examine the impacts of damages from the earthquake and the reconstruction process. The Prefectural Accounts only publishes the yearly base final demand. We need to estimate monthly base final demand by industry and region. Katayama and Yagi (2016) allocate the GPDP by month using RDEI, then calculate private consumption immediately following the earthquake using the Economy Watchers Survey.

Following their method, we calculate expenditures for each month by allocating nominal GPDP as in the Appendix Table 3 using RDEI by item in Fig. 2 and distributing by industry and region. Tourism consumption^13^ is measured by month, industry, and region using the Kumamoto Tourism Statistics.

Eligible expenditure items of final demand are (1) consumption of households, private Investment, (2) residential, (3) non-residential], (4) public Investment, and (5) tourism consumption. The target regions are Kumamoto City, Northern and Southern prefectures, and number of industries spans 105 sectors. The target period is 1 year before and after the earthquake (from April 2015 to May 2017).^14^ The benchmark of monthly expenditures is taken from FY2015, and if expenditures for each month in FY2016 are lower than in FY2015, the difference is considered a “decrease” due to earthquake damage; if they are higher, the difference is regarded as an “increase” caused by reconstruction demand. The sum of the final demand from 1 to 4 accounted for around 80% of the total GPDP.^15^

#### Estimation of monthly expenditure

1) Consumption of households

Multiplying the itemized expenditure according to purpose from the GPDP using the indexed spending by item of the FIES^16^ enables us to obtain household consumption by item and month. Since its monthly total is not equal to the estimated result of the monthly total in the preceding paragraph, the RAS method is used adjust the total.^17^ This total is distributed in each region using the regional ratios from population of the Basic Resident Registration. Then, the expenditure by item in each region is calculated with regional total by the composition ratio of the item category spending from the FIES. Thus, by rating itemized regional data in accordance with the industry classification in the IO table and converting them into producer prices, the expenditures by month, industry, and region are obtained.

2) Private residential investment

The monthly expenses are allocated to each region based on regional ratios of the construction floor area obtained from the BSS. Then, the gross regional domestic fixed capital formation (GRDFCF) (private sector) in 2016 and 2017 is calculated by multiplying the gross regional domestic fixed capital formation of the MRIO table by the growth rate of same item of the updated IO table for Japan in 2016 and 2017. These data are divided into residential and non-residential in accordance with the ratio obtained from the BSS and the Building Remodeling and Renewal Survey. Finally, the expenditure by industry is estimated by multiplying the composition ratio of residential parts by the spending by month and region.

3) Private non-residential investment

The private non-residential investment by month is distributed to each region using regional ratios taken from the acquisition amount of property, plant, and equipment in the Census of Manufacture.^18^ Then the expenditure by industry is calculated by multiplying the composition ratio of non-residential parts in 2) by the monthly expense and by region.

4) Public investment

The public investment by month is allocated to each region in accordance with regional ratios that obtained the investment expenses in the Settlement Cards. In addition, the GRDFCF (public sector) of the MRIO table is updated to 2016 and 2017 in a similar way as 2). Afterward, the expenditure by industry is valued by multiplying the composition ratio of them and the spending by month and region.

5) Tourism consumption

The monthly tourism consumption by region (MTCR) is estimated by multiplying the number of tourists (day trippers and lodgers, seasonally adjusted) by per capita consumption taken from the Kumamoto Tourism Statistics.^19^ Monthly tourism consumption by region and item are derived from multiplying MTCR by composition ratio by item taken from the Tourism Consumption Behavior Survey. This itemized consumption is converted to consumption by region and industry in a similar way to 1.

#### Change of the final demand

There are various interpretation of reconstruction and its effects. For example, return of certain economic indicators restore to before earthquake levels, or government spending to cope with the disaster. This study analyzes change in expenditure before and after the earthquake regardless of public or private, considering surplus above levels before the earthquake to reconstruction demand. The difference between the same months in FY2015 and FY2016 by item, region, and industry is obtained from the monthly expenditures estimated by the above procedure. If the amount of the expense in each month of FY 2016 is less than the previous fiscal year, the impact is treated as “decrease” due to earthquake damage. If the amount in FY2016 exceeds that of the previous fiscal year, the impact is treated as “increase” due to reconstruction demand.

### Analytical model 

To estimate economic ripple effects of the change in the final demand, the open Kumamoto MRIO table in 2015^20^ with endogenous import is used. For the estimation of the changes of production and value-added, the amount of the monthly expenditures change (producer prices^21^) are treated as given changes of the final demand ${\varvec{\Delta}}{\varvec{F}}$. The following equations are a MRIO equilibrium model^22^ and its derivation process. For details on the symbols (matrices, vectors, and these elements) used in the equations, see the Appendix (“Analytical model details” section).

Supply–demand balance on the MRIO table with endogenous import is

1 $${\varvec{X}}={\varvec{T}}{\varvec{A}}{\varvec{X}}+{\varvec{T}}{\varvec{F}}+{\varvec{E}}-\widehat{{\varvec{M}}}\left({{\varvec{T}}}_{{\varvec{L}}}{\varvec{A}}{\varvec{X}}+{{\varvec{T}}}_{{\varvec{L}}}{\varvec{F}}\right)$$

Solving (1) for ${\varvec{X}}$ gives

2 $${\varvec{X}}={\left[{\varvec{I}}-\left({\varvec{T}}-{\widehat{{\varvec{M}}}{\varvec{T}}}_{{\varvec{L}}}\right){\varvec{A}}\right]}^{-1}\cdot \left[\left({\varvec{T}}-{\widehat{{\varvec{M}}}{\varvec{T}}}_{{\varvec{L}}}\right){\varvec{F}}+{\varvec{E}}\right]$$

If reginal final demand changes by ${\varvec{\Delta}}{\varvec{F}}$, the induced regional domestic production is obtained as

3 $${\varvec{\Delta}}{\varvec{X}}={\left[{\varvec{I}}-\left({\varvec{T}}-{\widehat{{\varvec{M}}}{\varvec{T}}}_{{\varvec{L}}}\right){\varvec{A}}\right]}^{-1}\cdot \left({\varvec{T}}-\widehat{{\varvec{M}}}{{\varvec{T}}}_{{\varvec{L}}}\right){\varvec{\Delta}}{\varvec{F}}$$

Multiply the value-added ratio by ${\varvec{\Delta}}{\varvec{X}}$ to find the induced value-added accounted for the induced production:

4 $${\varvec{\Delta}}{\varvec{V}}=\widehat{{\varvec{v}}}{\varvec{\upgamma}}{\varvec{\Delta}}{\varvec{X}}$$

${\varvec{X}}$: Total regional domestic production vector; ${\varvec{A}}$: Input coefficient matrix; ${\varvec{F}}$: Final demand vector; ${\varvec{E}}$: Export vector; ${\varvec{T}}$: Inter-regional trade coefficient matrix; ${{\varvec{T}}}_{{\varvec{L}}}$: Intra-regional supply coefficient matrix; $\widehat{{\varvec{M}}}$: Import coefficient matrix; ${\varvec{I}}$: Identity matrix; ${\varvec{\Delta}}{\varvec{F}}$: Change of final demand vector; ${\varvec{\Delta}}{\varvec{X}}$: Induced production vector; ${\varvec{\Delta}}{\varvec{V}}$: Induced value-added vector; $\widehat{{\varvec{v}}}$: Value-added ratio matrix.^23^; ${\varvec{\gamma}}$: Converter matrix for consolidation of the industrial sector from 105 to 31; Number of Industrial sectors$: 105, 31$; Number of Regions: 4.

Assume a case where consumption is declining due to future uncertainty caused by the damage from the earthquake and the resulting stagnation of production activities. In this case, ${\varvec{\Delta}}{\varvec{F}}$ be negative, because expenditure is below the level of the same month of the previous fiscal year, and negative ripple effects (${\varvec{\Delta}}{\varvec{X}}$, ${\varvec{\Delta}}{\varvec{V}}$<0) be calculated. On the other hand, if expenditure is increasing due to reconstruction demand generated by lives back in order and restoration of infrastructure, ${\varvec{\Delta}}{\varvec{F}}$ be positive and positive ripple effects (${\varvec{\Delta}}{\varvec{X}}$, ${\varvec{\Delta}}{\varvec{V}}$>0) be estimated.

The monthly change vector of final demand by item, which is difference between same month FY2015-2016, is denoted by

5 $$\boldsymbol{\Delta }{{\varvec{F}}}_{{\varvec{m}}}^{{\varvec{k}}}={{\varvec{F}}}_{{\varvec{m}}\cdot{\varvec{F}}{\varvec{Y}}2016}^{{\varvec{k}}}-{{\varvec{F}}}_{{\varvec{m}}\cdot{\varvec{F}}{\varvec{Y}}2015}^{{\varvec{k}}}$$

Superscript $k$ represents the expenditure item, 1–5; subscript $m$ represents the month, 1–12. To analysis of impacts due to damage from earthquake or reconstruction demand separately divide change of final demand into increase and decrease:

6 $$\boldsymbol{\Delta }{{\varvec{F}}}_{{\varvec{m}}}^{{\varvec{k}}}=\boldsymbol{\Delta }{{\varvec{F}}}_{{\varvec{m}}}^{{\varvec{k}}\cdot +}+\boldsymbol{\Delta }{{\varvec{F}}}_{{\varvec{m}}}^{{\varvec{k}}\cdot -}$$

The superscript $+$ means that the negative element of ${\varvec{\Delta}}{\varvec{F}_{m}^{k}}$ is replaced by 0; $-$ means that the positive element is replaced by 0. Therefore, ${\varvec{\Delta}}{\varvec{F}}$ and ${\varvec{\Delta}}{\varvec{X}}$, ${\varvec{\Delta}}{\varvec{V}}$ in Eqs. (3) and (4) are replaced as follows:

7 $${\varvec{\Delta}}{{\varvec{X}}}_{{\varvec{m}}}^{{\varvec{k}}\cdot {\varvec{l}}}={\left[{\varvec{I}}-\left({\varvec{T}}-{\widehat{{\varvec{M}}}{\varvec{T}}}_{{\varvec{L}}}\right){\varvec{A}}\right]}^{-1}\cdot \left({\varvec{T}}-\widehat{{\varvec{M}}}{{\varvec{T}}}_{{\varvec{L}}}\right){\varvec{\Delta}}{{\varvec{F}}}_{{\varvec{m}}}^{{\varvec{k}}\cdot {\varvec{l}}}$$

8 $${\varvec{\Delta}}{{\varvec{V}}}_{{\varvec{m}}}^{{\varvec{k}}\cdot {\varvec{l}}}=\widehat{{\varvec{v}}}{\varvec{\upgamma}}{\varvec{\Delta}}{{\varvec{X}}}_{{\varvec{m}}}^{{\varvec{k}}\cdot {\varvec{l}}}$$

The superscript $l$ represents the mathematical symbol,$+$ or $-$. The induced production and the induced value-added obtained from Eqs. (7) and (8) are defined as the ripple effects for changes of final demand, by reconstruction demand in the case of $l=+$ and by earthquake damage in the case of $l=-$. This allows us to estimate the induced production and value-added by item and region due to the monthly change of expenditure caused by the earthquake.

## Results and discussion

The annual cumulative increase and decrease in expenditure and its ripple effects, which are estimated by methods of above section, by region in 1 year are shown in Table 2. In the following sections, we will examine this in detail, breaking it down by item, industry, and month for expenditure and induced value-added.

**Table 2:** Annual cumulative increase and decrease in expenditure and ripple effects

Region	Stage	(billion yen)		
		Annual cumulative		Net change
		Increase	Decrease	
Kumamoto Prefecture	Expenditure	648	− 592	56
	Domestic total demand	487	− 425	62
	Induced production	648	− 553	94
	Induced value-added	375	− 348	27
Kumamoto City	Expenditure	230	− 185	45
	Domestic total demand	182	− 167	15
	Induced production	253	− 230	23
	Induced value-added	153	− 152	1
Northern prefecture	Expenditure	246	− 297	− 51
	Domestic total demand	179	− 183	− 4
	Induced production	232	− 226	5
	Induced value-added	130	− 136	− 5
Southern prefecture	Expenditure	171	− 110	62
	Domestic total demand	127	− 75	51
	Induced production	163	− 98	66
	Induced value-added	92	− 61	31
Outside the prefecture	Expenditure	0	0	0
	Domestic total demand	121	− 118	4
	Induced production	406	− 326	81
	Induced value-added	181	− 153	28

The values for the annual cumulative total are the sum of the estimated for the amounts of increases or decreases by industry, item, and month in each stage and region. The annual cumulative increase and decrease in expenditure is the sum of ${\Sigma }_{k}{\Sigma }_{m}\boldsymbol{\Delta }{{\varvec{F}}}_{{\varvec{m}}}^{{\varvec{k}}\cdot{\varvec{l}}}$ by region. The annual cumulative increase and decrease in ripple effect is the sum of ${\Sigma }_{k}{\Sigma }_{m}\boldsymbol{\Delta }{{\varvec{X}}}_{{\varvec{m}}}^{{\varvec{k}}\cdot{\varvec{l}}}, {\Sigma }_{k}{\Sigma }_{m}\boldsymbol{\Delta }{{\varvec{V}}}_{{\varvec{m}}}^{{\varvec{k}}\cdot{\varvec{l}}}$ by region. The domestic total demand is the sum of ${\Sigma }_{k}{\Sigma }_{m}\left({\varvec{T}}-\widehat{{\varvec{M}}}{{\varvec{T}}}_{{\varvec{L}}}\right)\boldsymbol{\Delta }{{\varvec{F}}}_{{\varvec{m}}}^{{\varvec{k}}\cdot{\varvec{l}}}$ by region in the analytical model (7). However, tourism consumption is separately multiplied by the adjusted self-sufficiency rate. In addition, regional households consumption distributed by $\left({\varvec{T}}-\widehat{{\varvec{M}}}{{\varvec{T}}}_{{\varvec{L}}}\right)$ include tourism consumption, so some of the consumption of the two overlaps. Regarding these, household consumption may have been overestimated, because it is deemed difficult to make adjustments due to various constraints

### Change of final demand due to the earthquake

**Fig. 4:**
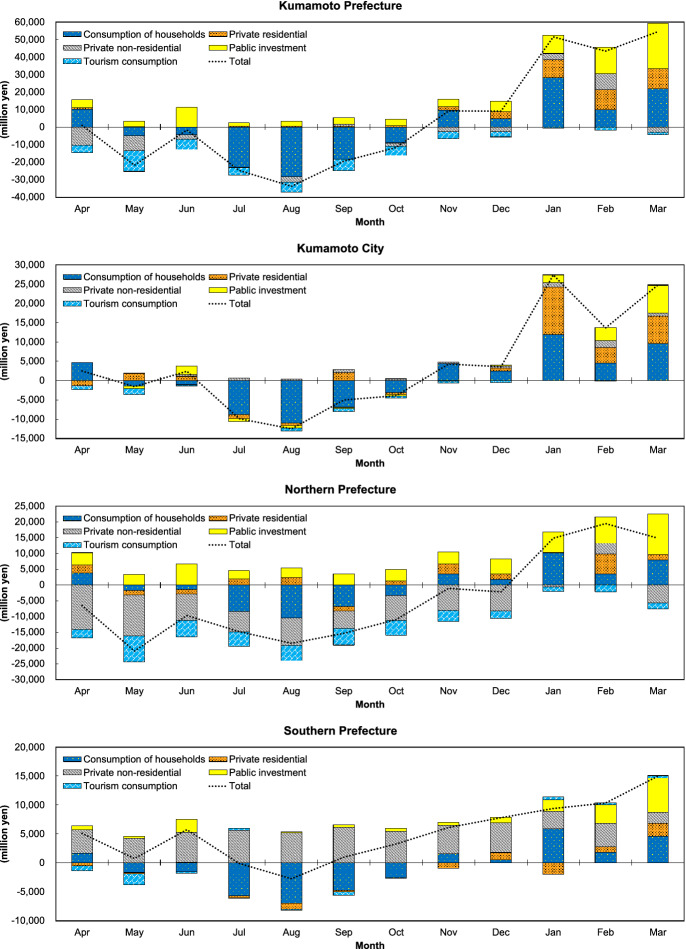
Change of the monthly final demand by region. The total is sum of the ${\sum }_{k}{\sum }_{l}{\varvec{\Delta}}{\varvec{F}_{m}^{k\cdot l}}$ by region and. The stacked bar by month and region of graphs shows amount of net change by item (the sum of ${\sum }_{l}{\varvec{\Delta}}{\varvec{F}_{m}^{k\cdot l}}$ by region). Therefore, sum of first or fourth quadrant is not equal to the cumulative increase or decrease in Table 2, which totals increase or decrease by item and industry, respectively

According to Table 2, the annual cumulative decrease of expenditure due to earthquake damage, which is derived from difference of the monthly spending in both fiscal years, is estimated at 592 billion yen. On the other hand, the annual cumulative increase in expenditure by reconstruction demand is calculated at 648 billion yen. These amounts accounted for 10.4% and 11.4% of the total GPDP in FY2015, respectively. Figure 4 shows transition of net change of monthly expenditure by region, and the vertical axis 0 of the figure signifies the level of expense in each month of FY2015.

Expenditure in the prefecture dropped down in from May to August 2016, then as the figure indicates, gradually recovered. From November 2016, it rose. Regarding household consumption, after a decrease for 6 months beginning in May, it recovered. The change in private residential investment was always positive except in June 2016 compared to FY2015. Private non-residential investment fell throughout 2016 except in September, with recovery in January and February of 2017. Public investment was positive throughout the fiscal year. In contrast, tourism consumption remained negative throughout FY2016.

Appendix Table 5 shows annual cumulative total of net change of monthly expenditure by item, region, and industry. In the entire region of the prefecture, increased amounts exceeded decreased ones in industries, such as construction, professional activities, transport equipment, and finance and insurance. These increased items are closely related to the consumption of households and private investment. On the other hand, in real estate, transport and postal service, and other service activities, increased expenditure amounts were less than the decreased amounts.

The impacts on each region are as follows. In Kumamoto City, where earthquake damage was moderate, annual cumulative drop and rise was recorded at 185 billion yen and 230 billion yen, respectively. The monthly expenditure exceeded the previous year's level immediately after the earthquake due to a temporary increase in household consumption, but then plunged and stagnated for several months. It almost recovered in November 2016 and subsequently experienced a significant increase due to the growth of private residential investment and public investment. It is thought that the increase in household consumption immediately after the disaster was an emergency response to the numerous injuries and damage to housing, with a sharp drop due to the backlash and uncertainty about the future, followed by an increase in full-fledged reconstruction demand.

In the northern part of the prefecture, where main infrastructures were severely damaged, the annual cumulative decrease and increase were 297 billion yen and 246 billion yen, respectively. Both amounts were the largest among the three regions. Although public investment was expanded to overcome the damage, total spending did not recover in 2016 because of difficulty in restoring private non-residential investment and household and tourism consumption. Consequently, noticeable decreases appeared in accommodations and food service activities, and general machinery. The notable weakness in non-residential investment and tourism consumption indicates the extent of the damage to people and buildings and its harmful rumors. In line with the scale of the damage, public investment also expanded, with the largest net increase, but it did not cover even half of the decline in other expenditures.

In the southern part of the prefecture, where the amount of damage suffered was small, the recorded annual cumulative decline was 110 billion yen and the annual cumulative increase was 171 billion yen. Both amounts were the smallest among the regions, but the net change of expenditure was the biggest. Although this area experienced slightly decreased expenditures in July and August 2016 due to a decline in household consumption, increased spending on non-residential and public investment pushed the other months spending above the previous fiscal year’s level. However, the annual cumulative total of residential investment was the only net decrease in the prefecture, due in part to the extremely low number of housing collapses. Looking at industry, as a result of the increased spending, the region conspicuously gained in the activities of construction and professional service.

### The ripple effects

It is estimated that the annual cumulative decrease in prefecture spending pushed down final demand by 425 billion yen and production by 553 billion yen in the prefecture as a whole. On the other hand, the annual cumulative increase in expenditure boosted final demand by 487 billion yen and induced 648 billion yen gain in production in the prefecture as a whole. As a result, prefectural production is estimated to have grown by 94 billion yen in 2016. This amount accounts for 19.9% of the increase in output of the prefectural accounts in FY2015-2016.

**Fig. 5:**
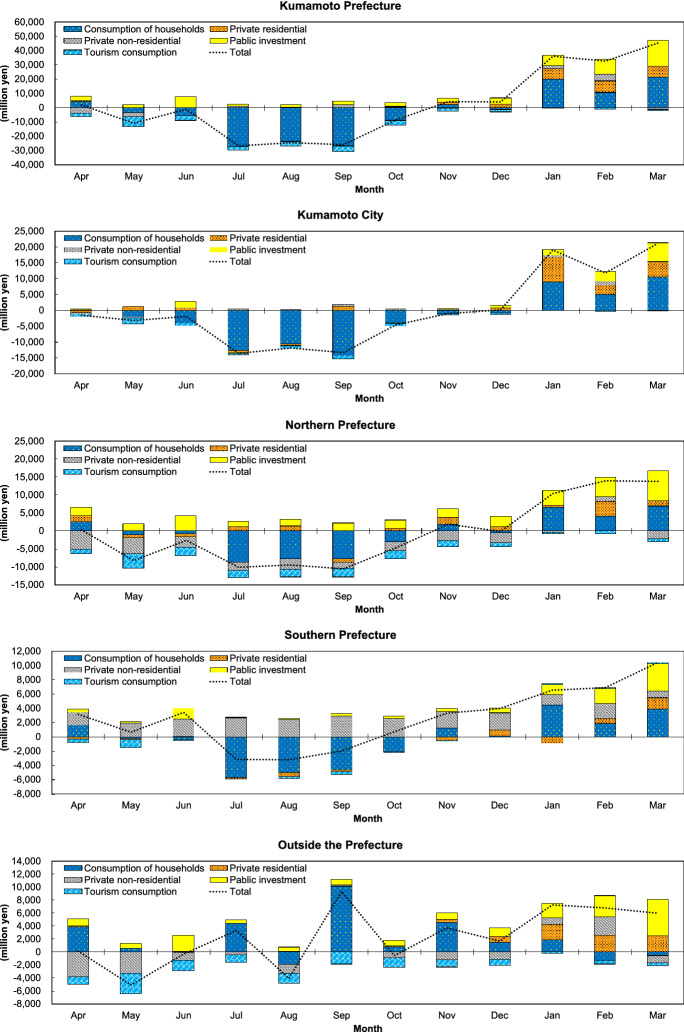
Net change of the monthly induced value-added by region. The total is the sum of ${\sum }_{k}{\sum }_{l}{\varvec{\Delta}}{\varvec{V}_{m}^{k\cdot l}}$ by region. The stacked bar by month and region of graphs shows amount of net change by item (the sum of ${\sum }_{l}{\varvec{\Delta}}{\varvec{V}_{m}^{k\cdot l}}$ by region). Therefore, sum of first or fourth quadrant is not equal to the cumulative increase or decrease in Table 2, which totals increase or decrease by item and industry, respectively

In addition, annual cumulative value-added loss was 348 billion yen, and annual cumulative gain was 375 billion yen; that resulted in a net gain of 27 billion yen. This net gain was equivalent to 10.9% of the increase in GPDP of 246 billion yen over the same period. The change of final demand are calculated based on GPDP (expenditure). Therefore, the Induced value-added caused by those fit^24^ the range of GPDP (production). In other words, the induced value-added is a part of GPDP, the ratio indicates the extent to which the induced value-added cover GPDP. Figure 5 shows the transition of the net change of induced value-added due to the monthly change of expenditure by region. Appendix Table 6 shows the annual cumulative total of net change of monthly induced value-added by item, region, and industry.

According to Table 2, net change of the induced value-added of Kumamoto City, northern, and southern prefecture is 1.4 billion yen, − 5.3 billion yen, and 30.8 billion yen, which is equivalent to 1.5%, − 3.8%, and 108.4% to the total amount of the gross regional domestic product (GRDP) change in each region over the same period, respectively.^25^

While expenditure in the northern prefecture experienced the most decrease among the regions, drop in imports due to the decline in the non-residential investment demand. Consequently, net change in production became positive in this area. Comparing Figs. 4 and 5, shows that the decline in non-residential investment has narrowed and that the net change of induced value-added brought about by expenditures in April and November has become positive. Although the net change of annual cumulative induced value-added remained negative. In Kumamoto City, where the increased expenditures significantly exceeded decreased expenditures, the fall in exports due to decline in demand outside the city, and rise in imports due to leakage of demand to outside the city. Therefore, the annual cumulative increase and the annual cumulative decrease in regional domestic demand were at a same level. The net changes of the induced value-added due to the monthly change of expenditure were mostly negative within 2016, and their annually total became significantly small. In the south of the prefecture, where net increase in expenditure was largest, imports gained due to leakage of the private non-residential investment demand. Thereby, net change in annually induced production and value-added remained positive and was the largest in the prefecture, though the net decrease in induced value-added due to the monthly change of expenditure ware expanded. Regarding regions outside the prefecture, trade with the prefecture generated an increase and decrease in regional domestic demand same level as the southern prefecture. However, the cumulative increase in the ripple effects far outweighed the cumulative decrease; the net increase of value-added were more than that of the Kumamoto Prefecture. Transactions of the net change of induced value-added are roughly similar to those in the prefecture, although July and September show significantly different movements due to the influx of manufacturing-related demand.

By region and industry (Appendix Table 6), Kumamoto City has seen severe negative outcomes in other service activities and real estate, while construction and professional activities have seen positive outcomes, with the investments offsetting the decrease in the consumptions. In the northern prefecture, there has been a marked negative impact in sectors of relevance to non-residential investment and tourism, but a large growth in construction by public investment has compensated for the drop to some extent. In the southern prefecture, the sectors of relation to household and residential experienced relatively small declines, and construction and professional activities related to non-residential and public investment greatly exceeded these. Outside the prefecture, the value-added has decreased in tourism consumption and non-residential investment-related sectors. On the contrary, it has increased markedly in transport equipment and professional activities, with household consumption making up for most of the decline.

## Conclusions

This study investigated the increase and decrease in expenditure due to the damage and reconstruction demand caused by the Kumamoto earthquake, and analyzed the impact of the earthquake on production and added value in and outside of the prefecture using the MRIO table.

Our findings indicate that earthquake damage caused an annual cumulative decrease in expenditures of 592 billion yen and value-added losses of 348 billion yen in the prefecture as a whole. Reconstruction demand led to an annual cumulative increase in spending of 648 billion yen and a value-added gain of 375 billion yen in the prefecture. The net increase in value-added of 27 billion yen, which difference of increase and decrease in value-added inducement, accounted for 10.9% of the total GPDP gain during the same period. Looking at monthly transitional changes, the expenditure level was less than the previous fiscal year’s level during several months due to the reduction of household consumption, tourism consumption, and private non-residential investment. A few months after the earthquake, the level recovered more than that of the previous fiscal year because of the rapid expansion of private residential and public investment. In the northern prefecture, where damage was particularly bad, spending fell sharply due to a drop in consumption and non-residential investment, while an expansion of production through residential and public investment compensated for these losses to some extent. In Kumamoto City, where the damage was moderate, though an increase in expenditures was significantly higher than the decrease, net gain in value-added was significantly small due to leakage of these demand to outside the city. In the southern prefecture, where damage was relatively mild, both expenditure and value-added loss exceeded the previous fiscal year’s levels for many months due to the expansion of non-residential investment, and the excess of the increase was the largest among the three regions. Outside the prefecture experienced increase and decrease in demand about the same as that of southern prefecture through trade, and the net rise in value-added greater than that of the prefecture.

In this way, the monthly increase and decrease in expenditures and its induced production and value-added owing to the Kumamoto earthquake was very large, and the damage to the prefecture’s economy was enormous. Recovery was achieved in a short period of time by virtue of reconstruction-related demand that exceeded the economic damage. However, this is only true for the prefecture's economy as a whole, a delay in restoration was observed in areas, where damage was concentrated and in industries with weak links to reconstruction-related demand. Our results also show that the change in expenditure within the prefecture had significant impact on the other parts of Japan. To our best knowledge this is the first time to use the monthly data by region, item, and industry for the investigation of the economic impacts of the Kumamoto earthquake.

However, there are several issues to be considered. As a final demand, government consumption and imports/exports which are not included in the scope of this study, have a considerable weight in the gross prefectural domestic product. Thus, the impact of changes in these items is expected to be larger than our estimation. In addition, a comparison of the estimation results with the prefectural and municipal accounts suggests a bias in the allocation of expenditure to industries and regions.^26^ Although supply constraints due to the earthquake may bring about changes in the economic structure, this study has not been able to take this into account. Moreover, this study did not consider the financial burden of subsidies, restoration investments, and other forms of reconstruction assistance. If these are financed by taxes or government bonds, it will be a tax burden on the people in the future and is expected to have a negative impact on the economy. Besides, as indicated in the Background and Results section, not everything was resolved during the targeted period. Although lives back in order and restoring infrastructure after a great disaster takes a long period of time, which was not covered in this analysis. All of these issues are left for further study.

## Data Availability

The data sets generated and/or analyzed during the current study are available from the corresponding author on reasonable request. Statistics used in this paper can be obtained at the following website of public offices. Cabinet Office: National Accounts, Gross Domestic Product, Regional Domestic Expenditure Index; Ministry of Economy, Trade and Industry: Updated Input–Output Table, Census of Manufacture, Indices of Industrial Production; Ministry of Land, Infrastructure, Transport and Tourism: Building Starts Statistics, Building Remodeling and Renewal Survey, Tourism Consumption Behavior Survey; Ministry of Internal Affairs and Communications: Input Output Table for Japan, Population of the Basic Resident Registration, Settlement Cards, Family Income and Expenditure Survey, Economic Census; Kumamoto Prefectural Office: Input–Output Table for Kumamoto Prefecture, Prefectural Accounts, Municipal Accounts, Gross Prefectural Domestic Product, Gross Regional Domestic Product, Kumamoto Tourism Statistics.

## Appendix

See Tables 3, 4, 5, 6﻿.

**Table 3:** Areas on the MRIO table

Areas	Municipalities				
Northern prefecture	Kikuchi City	Koshi City	Ozu Town	Kikuyo Town	Arao Town
	Nagasu Town	Yamaga City	Aso City	Minamioguni Town	Oguni Town
	Tamana City	Gyokuto Town	Izumi Town	Nankan Town	Ubuyama Village
	Takamori Town	Minamiaso Village	Nishihara Village	Uto City	Uki City
	Misato Town	Mihune Town	Kashima Town	Mashiki Town	Kosa Town
	Yamato Town				
Kumamoto City	Kumamoto City				
Southern prefecture	Yatsushiro City	Hikawa Town	Minamata City	Ashikita Town	Tsunagi Town
	Yunomae Town	Mizukami Village	Sagara Village	Itsuki Village	Yamae Village
	Hitoyoshi City	Nisiki Town	Asagiri Town	Taragi Town	Kuma Village
	Amakusa City	Kamiamakusa City	Reihoku Town		
Outside the prefecture	Other parts of Japan

**Table 4:** Nominal GPDP (expenditure approach)

	(billion yen)		
	FY2015	FY2016	Difference
Private final consumption expenditure	3477	3474	− 3
Final consumption expenditure of households	3386	3383	− 3
Food and non-alcoholic beverages	575	579	5
Alcoholic beverages, tobacco	93	92	− 0
Clothes and shoes	136	122	− 14
Housing, water, electricity, gas and other fuels	796	777	− 19
Furnishings, household equipment, maintenance of the house	159	160	1
Health	106	103	− 3
Transport	363	382	19
Communication (PTT services)	146	149	4
Recreation and culture	287	282	− 6
Education	62	61	− 1
Restaurants and hotels	273	283	10
Other goods and services	390	392	3
Final consumption expenditure of private non-profit institutions serving households	91	91	0
Government final consumption expenditure	1590	1720	130
Gross capital formation	1485	1536	50
Gross fixed capital formation			
Private sectors			
Residential	181	223	42
Non-residential	890	869	− 21
Public sectors			
Residential	5	3	− 2
Non-residential	30	28	− 2
General government	341	438	96
Changes in inventories	38	− 24	− 63
Net exports of goods and services, statistical discrepancy	− 867	− 789	78
Gross prefectural domestic product (expenditure approach)	5686	5941	256

The Kumamoto prefectural account

**Table 5:** Change of final demand (annual cumulative total, 31 sectors)

Region	Rank	Industries	Total	(million yen)				
				Net change of expenditure (annual cumulative total)				
				1)Consumption of Households	2) Residential investment	3) Non-residential investment	4) Public investment	5) Tourism consumption
Kumamoto Prefecture	1	Construction	123,651	0	41,950	6443	75,259	0
	2	Finance and insurance	41,246	41,246	0	0	0	0
	3	Transport equipment	30,222	29,118	0	− 227	1,331	0
	4	Electricity, gas and water supply and waste management service	18,334	18,334	0	0	0	0
	5	Professional, scientific and technical activities	14,640	4768	0	− 920	10,941	− 148
	~	Others	− 28,079	22,388	0	− 15,129	2,793	− 38,131
	27	Information and communication electronics equipment	− 10,905	− 1,413	0	− 11,264	1,841	− 69
	28	Other manufacturing	− 11,372	− 10,158	0	− 309	165	− 1,070
	29	Other service activities	− 31,146	− 23,967	0	0	0	− 7,179
	30	Transport and postal services	− 37,517	− 30,079	0	− 136	221	− 7,523
	31	Real estate	− 53,198	− 53,267	0	68	0	0
	32	Total	55,876	− 3,029	41,950	− 21,475	92,551	− 54,121
Kumamoto City	1	Construction	40,134	0	25,662	3,843	10,628	0
	2	Finance and insurance	17,205	17,205	0	0	0	0
	3	Transport equipment	12,059	11,870	0	39	151	0
	4	Electricity, gas and water supply and waste management service	7730	7730	0	0	0	0
	5	Accommodation and food service activities	7327	9813	0	0	0	− 2486
	~	Others	9330	5928	0	3162	1562	− 1322
	27	Textile products	− 1803	− 1738	0	9	0	− 74
	28	Other manufacturing	− 4020	− 3973	0	9	20	− 76
	29	Other service activities	− 10,132	− 9303	0	0	0	− 829
	30	Transport and postal services	− 12,303	− 11,791	0	122	32	− 666
	31	Real estate	− 20,158	− 20,214	0	56	0	0
	32	Total	45,370	5528	25,662	7239	12,393	− 5453
Northern Prefecture	1	Construction	49,874	0	16,955	− 17,523	50,442	0
	2	Finance and insurance	15,216	15,216	0	0	0	0
	3	Transport equipment	9394	10,756	0	− 2,262	900	0
	4	Electricity, gas and water supply and waste management service	6762	6762	0	0	0	0
	5	Agriculture	78	871	0	− 403	0	− 390
	~	Others	− 52,233	− 3829	0	− 47,116	10,960	− 12,249
	27	Accommodation and food service activities	− 12,940	8548	0	0	0	− 21,488
	28	General-purpose, production and business oriented machinery	− 13,961	30	0	− 14,610	619	0
	29	Other service activities	− 15,084	− 8,867	0	0	0	− 6217
	30	Transport and postal services	− 18,543	− 11,128	0	− 1,367	157	− 6204
	31	Real estate	− 19,776	− 19,728	0	− 47	0	0
	32	Total	− 51,213	− 1370	16,955	− 83,329	63,078	− 46,549
Southern Prefecture	1	Construction	33,644	0	− 668	20,123	14,189	0
	2	Professional, scientific and technical activities	18,931	1025	0	16,002	1820	83
	3	Finance and insurance	8825	8825	0	0	0	0
	4	Transport equipment	8768	6492	0	1996	280	0
	5	General-purpose, production and business oriented machinery	5436	18	0	5240	179	0
	~	Others	14,137	6189	0	8787	489	− 1327
	27	Information and communications	− 837	− 1029	0	91	60	40
	28	Other manufacturing	− 1319	− 2427	0	1206	31	− 130
	29	Other service activities	− 5930	− 5797	0	0	0	− 133
	30	Transport and postal services	− 6671	− 7160	0	1110	32	− 652
	31	Real estate	− 13,265	− 13,324	0	59	0	0
	32	Total	61,719	− 7188	− 668	54,614	17,080	-2119

Net change of expenditure by item $={\sum }_{l}{\sum }_{m}{\varvec{\Delta}}{\varvec{F}_{m}^{k\bullet l}}$, total $={\sum }_{k}{\sum }_{l}{\sum }_{m}{\varvec{\Delta}}{\varvec{F}_{m}^{k\bullet l}}$. Moreover, consolidated industrial sectors from 105 to 31. The top and bottom 5 sectors are displayed by region

**Table 6:** Change of induced value-added (annual cumulative total, 31 sectors)

Region	Rank	Industries	Total	(million yen)				
				Net change of induced value-added (annual cumulative total)				
				1) Consumption of Households	2) Residential investment	3) Non-residential investment	4) Public investment	5) Tourism consumption
Kumamoto Prefecture	1	Construction	57,646	− 179	19,635	3011	35,249	− 69
	2	Finance and insurance	19,404	18,544	341	25	834	− 340
	3	Professional, scientific and technical activities	17,009	1546	3222	− 562	14,483	− 1681
	4	Electricity, gas and water supply and waste management service	7746	8047	219	− 14	553	− 1059
	5	Non-metallic mineral products	2791	− 57	721	147	1997	− 17
	~	Others	905	589	1651	− 724	3591	− 4201
	27	Wholesale and retail trade	− 2801	− 1523	1210	− 794	2417	− 4110
	28	Accommodation and food service activities	− 3315	7310	0	− 0	0	− 10,625
	29	Transport and postal services	− 10,808	− 12,183	1790	− 19	3928	− 4323
	30	Other service activities	− 16,504	− 12,960	323	34	606	− 4508
	31	Real estate	− 45,271	− 45,813	412	8	842	− 719
	32	Total	26,802	− 36,679	29,524	1111	64,499	− 31,653
Kumamoto City	1	Construction	18,652	− 118	12,008	1800	4981	− 19
	2	Finance and insurance	12,947	12,401	263	28	463	− 208
	3	Professional, scientific and technical activities	7046	76	2329	1187	4291	− 836
	4	Accommodation and food service activities	1917	3874	0	− 0	0	− 1956
	5	Electricity, gas and water supply and waste management service	1719	1725	51	10	63	− 130
	~	Others	790	6	1210	121	1305	− 1851
	27	Information and communications	− 430	− 918	135	76	644	− 367
	28	Education	− 601	− 206	11	0	18	− 423
	29	Transport and postal services	− 5862	− 6537	989	183	786	− 1283
	30	Other service activities	− 7948	− 7757	170	30	162	− 554
	31	Real estate	− 26,853	− 27,309	323	59	499	− 424
	32	Total	1379	− 24,763	17,489	3494	13,212	− 8053
Northern Prefecture	1	Construction	23,248	− 37	7937	− 8231	23,621	− 43
	2	Finance and insurance	3512	3408	62	− 103	245	− 100
	3	Non-metallic mineral products	1839	− 10	473	− 173	1558	− 8
	4	Electricity, gas and water supply and waste management service	1030	1420	60	− 126	194	− 518
	5	Fabricated metal products	740	87	403	− 265	531	− 17
	~	Others	− 5633	288	938	− 4676	2595	− 4779
	27	Professional, scientific and technical activities	− 3732	792	805	− 12,638	8071	− 762
	28	Transport and postal services	− 4836	− 3793	738	− 1562	2443	− 2663
	29	Accommodation and food service activities	− 6282	2145	0	− 0	0	− 8426
	30	Other service activities	− 6505	− 2970	129	− 176	329	− 3818
	31	Real estate	− 8726	− 8500	82	− 312	269	− 266
	32	Total	− 5346	− 7169	11,626	− 28,263	39,858	− 21,398
Southern Prefecture	1	Construction	15,746	− 24	− 310	9441	6646	− 7
	2	Professional, scientific and technical activities	13,695	678	89	10,890	2122	− 83
	3	Electricity, gas and water supply and waste management service	4997	4902	108	102	296	− 411
	4	Finance and insurance	2945	2736	15	99	126	− 32
	5	Wholesale and retail trade	1107	− 531	21	1552	346	− 280
	~	Others	4817	2293	392	1919	979	− 765
	27	Transport and postal services	− 110	− 1853	63	1360	698	− 378
	28	Human health and social work activities	− 315	− 245	0	0	0	− 71
	29	Information and communications	− 369	− 465	1	75	29	− 10
	30	Other service activities	− 2051	− 2233	24	180	114	− 136
	31	Real estate	− 9692	− 10,005	7	261	73	− 29
	32	Total	30,770	− 4746	410	25,880	11,429	− 2,202
Outside the Prefecture	1	Transport equipment	11,443	11,161	60	− 117	491	− 153
	2	Professional, scientific and technical activities	7084	1607	2216	− 610	5734	− 1864
	3	Finance and insurance	6799	6454	257	− 110	599	− 401
	4	Basic metal	3798	1683	964	− 551	1815	− 113
	5	Accommodation and food service activities	2595	2604	0	− 0	0	− 9
	~	Others	9712	7910	4960	− 3336	9538	− 9359
	27	Textile products	− 633	− 583	28	− 22	45	− 102
	28	Other service activities	− 1306	− 1542	162	− 62	315	− 179
	29	Information and communication electronics equipment	− 1679	− 186	11	− 1809	322	− 16
	30	General-purpose, production and business oriented machinery	− 2368	407	190	− 3487	582	− 61
	31	Transport and postal services	− 7116	− 5522	512	− 333	1119	− 2892
	32	Total	28,329	23,993	9360	− 10,437	20,560	− 15,148

Net change of expenditure by item $={\sum }_{l}{\sum }_{m}{\varvec{\Delta}}{\varvec{V}_{m}^{k\bullet l}}$, total $={\sum }_{k}{\sum }_{l}{\sum }_{m}{\varvec{\Delta}}{\varvec{V}_{m}^{k\bullet l}}$. Moreover, consolidated industrial sectors from 105 to 31. The top and bottom 5 sectors are displayed by region

### Analytical model details

The following based on the Kumamoto MRIO table in 2015. The number of industrial sector and regions is 105 and 4. Superscript *r* and *s* can be *C, N, S*, and *O* and represent Kumamoto City, Northern prefecture, Southern prefecture, and Outside of the prefecture, respectively. Subscript *i* and *j* represent industrial sector and can be from 1 to 105.

- Supply-demand balance on the MRIO table with endogenous import (repost)
  9 $${\varvec{X}}={\varvec{T}}{\varvec{A}}{\varvec{X}}+{\varvec{T}}{\varvec{F}}+{\varvec{E}}-\widehat{{\varvec{M}}}\left({{\varvec{T}}}_{{\varvec{L}}}{\varvec{A}}{\varvec{X}}+{{\varvec{T}}}_{{\varvec{L}}}{\varvec{F}}\right)$$
- MRIO Equilibrium Model (repost)
  10 $${\varvec{X}}={\left[{\varvec{I}}-\left({\varvec{T}}-\widehat{{\varvec{M}}}{{\varvec{T}}}_{{\varvec{L}}}\right){\varvec{A}}\right]}^{-1}\cdot \left[\left({\varvec{T}}-\widehat{{\varvec{M}}}{{\varvec{T}}}_{{\varvec{L}}}\right){\varvec{F}}+{\varvec{E}}\right]$$
- The Ripple Effect (repost)
  11 $${\varvec{\Delta}}{\varvec{X}}={\left[{\varvec{I}}-\left({\varvec{T}}-\widehat{{\varvec{M}}}{{\varvec{T}}}_{{\varvec{L}}}\right){\varvec{A}}\right]}^{-1}\cdot \left({\varvec{T}}-\widehat{{\varvec{M}}}{{\varvec{T}}}_{{\varvec{L}}}\right){\varvec{\Delta}}{\varvec{F}}$$
  12 $${\varvec{\Delta}}{\varvec{V}}=\widehat{{\varvec{v}}}{\varvec{\upgamma}}{\varvec{\Delta}}{\varvec{X}}$$
- Symbols and Elements

Regional Domestic Production vector

$${\varvec{X}}=\left[\begin{array}{c}{{\varvec{X}}}^{{\varvec{C}}}\\ {{\varvec{X}}}^{{\varvec{N}}}\\ {{\varvec{X}}}^{{\varvec{S}}}\\ {{\varvec{X}}}^{{\varvec{O}}}\end{array}\right],\boldsymbol{ }{{\varvec{X}}}^{{\varvec{r}}}=\left[\begin{array}{c}{X}_{1}^{r}\\ \vdots \\ {X}_{n}^{r}\end{array}\right]$$

${X}_{i}^{r}$: Domestic production of *i*-goods in *r*-region.

Input Coefficient matrix

$$\varvec{A}=\left[ \begin{array} {cccc}  {{\varvec{A}}^{\varvec{C}}} & \boldsymbol{0} & \boldsymbol{0} & \boldsymbol{0}  \\   \boldsymbol{0} & {{\varvec{A}}^{\varvec{N}}} & \boldsymbol{0} & \boldsymbol{0}  \\   \boldsymbol{0} & \boldsymbol{0} & {{\varvec{A}}^{\varvec{S}}} & \boldsymbol{0}  \\   \boldsymbol{0} & \boldsymbol{0} & \boldsymbol{0} & {{\varvec{A}}^{\varvec{O}}}  \\ \end{array} \right], \, \boldsymbol{ }{{\varvec{A}}}^{{\varvec{r}}}=\left[\begin{array}{ccc}{a}_{11}^{r}& \dots & {a}_{1n}^{r}\\ \vdots & \ddots & \vdots \\ {a}_{n1}^{r}& \dots & {a}_{nn}^{r}\end{array}\right]$$

${a}_{ij}^{r}=\frac{{x}_{ij}^{r}}{{X}_{j}^{r}}$: Input coefficient between *i*-sector and *j*-sector in *r*-region.

${x}_{ij}^{r}$: Intermediate transactions between *i*-sector and *j*-sector in *r*-region.

Final Demand vector

$${\varvec{F}}=\left[\begin{array}{c}{{\varvec{F}}}^{{\varvec{C}}}\\ {{\varvec{F}}}^{{\varvec{N}}}\\ {{\varvec{F}}}^{{\varvec{S}}}\\ {{\varvec{F}}}^{{\varvec{O}}}\end{array}\right],\boldsymbol{ }{{\varvec{F}}}^{{\varvec{r}}}=\left[\begin{array}{c}{F}_{1}^{r}\\ \vdots \\ {F}_{n}^{r}\end{array}\right]$$

${F}_{i}^{r}$: Final demand of *i*-goods in *r*-region.

Exports vector

$${\varvec{E}}=\left[\begin{array}{c}{{\varvec{E}}}^{{\varvec{C}}}\\ {{\varvec{E}}}^{{\varvec{N}}}\\ {{\varvec{E}}}^{{\varvec{S}}}\\ {{\varvec{E}}}^{{\varvec{O}}}\end{array}\right],\boldsymbol{ }{{\varvec{E}}}^{{\varvec{r}}}=\left[\begin{array}{c}{E}_{1}^{r}\\ \vdots \\ {E}_{n}^{r}\end{array}\right]$$

${E}_{i}^{r}$: Export of *i*-goods in *r*-region.

Inter-regional Trade Coefficient matrix

$${\varvec{T}}=\left[\begin{array}{cc}\begin{array}{cc}{{\varvec{T}}}^{{\varvec{C}}{\varvec{C}}}& {{\varvec{T}}}^{{\varvec{C}}{\varvec{N}}}\\ {{\varvec{T}}}^{{\varvec{N}}{\varvec{C}}}& {{\varvec{T}}}^{{\varvec{N}}{\varvec{N}}}\end{array}& \begin{array}{cc}{{\varvec{T}}}^{{\varvec{C}}{\varvec{S}}}& {{\varvec{T}}}^{{\varvec{C}}{\varvec{O}}}\\ {{\varvec{T}}}^{{\varvec{N}}{\varvec{S}}}& {{\varvec{T}}}^{{\varvec{N}}{\varvec{O}}}\end{array}\\ \begin{array}{cc}{{\varvec{T}}}^{{\varvec{S}}{\varvec{C}}}& {{\varvec{T}}}^{{\varvec{S}}{\varvec{N}}}\\ {{\varvec{T}}}^{{\varvec{O}}{\varvec{C}}}& {{\varvec{T}}}^{{\varvec{O}}{\varvec{N}}}\end{array}& \begin{array}{cc}{{\varvec{T}}}^{{\varvec{S}}{\varvec{S}}}& {{\varvec{T}}}^{{\varvec{S}}{\varvec{O}}}\\ {{\varvec{T}}}^{{\varvec{O}}{\varvec{S}}}& {{\varvec{T}}}^{{\varvec{O}}{\varvec{O}}}\end{array}\end{array}\right],\boldsymbol{ }{{\varvec{T}}}^{{\varvec{r}}{\varvec{s}}}=\left[\begin{array}{ccc}{t}_{1}^{rs}& \dots & 0\\ \vdots & \ddots & \vdots \\ 0& \dots & {t}_{n}^{rs}\end{array}\right]$$

$${t}_{i}^{rs}=\frac{\mathrm{Imports \,from\, }r-\mathrm{region \,in\, }s-\mathrm{region}}{\mathrm{Total \,domestic \,demand \,in \,}s-\mathrm{region}} =\frac{{N}_{i}^{rs}}{{{{\Sigma }_{j}a}_{ij}^{s}X}_{j}^{s}+{F}_{i}^{s}} r\ne s$$

: Inter-regional trade coefficient of *i*-goods from *r*-region to *s*-region

${t}_{i}^{ss}=1-{\Sigma }_{r}{t}_{i}^{rs}$: Intra-regional supply coefficient of *i*-goods in *s*-region.

Intra-regional Supply Coefficient matrix

$$\varvec{T}_{\varvec{L}}=\left[ \begin{array} {llll}  {{\varvec{T}}^{\varvec{CC}}} & \boldsymbol{0} & \boldsymbol{0} & \boldsymbol{0}  \\   \boldsymbol{0} & {{\varvec{T}}^{\varvec{NN}}} & \boldsymbol{0} & \boldsymbol{0}  \\   \boldsymbol{0} & \boldsymbol{0} & {{\varvec{A}}^{\varvec{SS}}} & \boldsymbol{0}  \\   \boldsymbol{0} & \boldsymbol{0} & \boldsymbol{0} & {{\varvec{A}}^{\varvec{OO}}}  \\ \end{array} \right], \,\boldsymbol{ }{{\varvec{T}}}^{{\varvec{s}}{\varvec{s}}}=\left[\begin{array}{ccc}{t}_{1}^{ss}& \dots & 0\\ \vdots & \ddots & \vdots \\ 0& \dots & {t}_{n}^{ss}\end{array}\right]$$

Import Coefficient matrix

$$\varvec{\widehat{M}}=\left[ \begin{array} {llll}  \varvec{\widehat{M}}^{\varvec{C}} & \boldsymbol{0} & \boldsymbol{0} & \boldsymbol{0}  \\   \boldsymbol{0} & \varvec{\widehat{M}}^{\varvec{N}} & \boldsymbol{0} & \boldsymbol{0}  \\   \boldsymbol{0} & \boldsymbol{0} & \varvec{\widehat{M}}^{\varvec{S}} & \boldsymbol{0}  \\   \boldsymbol{0} & \boldsymbol{0} & \boldsymbol{0} & \varvec{\widehat{M}}^{\varvec{O}}  \\ \end{array} \right], \, \boldsymbol{ }{\widehat{{\varvec{M}}}}^{{\varvec{r}}}=\left[\begin{array}{ccc}{m}_{1}^{r}& \dots & 0\\ \vdots & \ddots & \vdots \\ 0& \dots & {m}_{n}^{r}\end{array}\right]$$

$${m}_{i}^{r} =\frac{{M}_{i}^{r}}{{{{\Sigma }_{j}{t}_{i}^{rr}a}_{ij}^{r}X}_{j}^{r}+{t}_{i}^{rr}{F}_{i}^{r}}$$

: Import coefficient of *i*-goods from abroad to *r*-region.

${M}_{i}^{r}$: Imports of *i*-goods from abroad to *r*-region.

Identity matrix: $${\varvec{I}}$$

Zero matrix: **0**

Change of Final Demand vector

$${\varvec{\Delta}}{\varvec{F}}=\left[\begin{array}{c}{\varvec{\Delta}} {{\varvec{F}}}^{{\varvec{C}}}\\ {{\varvec{\Delta}}{\varvec{F}}}^{{\varvec{N}}}\\ {\varvec{\Delta}} {{\varvec{F}}}^{{\varvec{S}}}\\ {{\varvec{\Delta}}{\varvec{F}}}^{{\varvec{O}}}\end{array}\right],\boldsymbol{ }{{\varvec{\Delta}}{\varvec{F}}}^{{\varvec{r}}}=\left[\begin{array}{c}{\Delta F}_{1}^{r}\\ \vdots \\ {\Delta F}_{n}^{r}\end{array}\right]$$

${\Delta F}_{i}^{r}$: Change of final demand of *i*-goods in *r*-region.

Induced Production vector

$${\varvec{\Delta}}{\varvec{X}}=\left[\begin{array}{c}{{\varvec{\Delta}}{\varvec{X}}}^{{\varvec{C}}}\\ {{\varvec{\Delta}}{\varvec{X}}}^{{\varvec{N}}}\\ {{\varvec{\Delta}}{\varvec{X}}}^{{\varvec{S}}}\\ {{\varvec{\Delta}}{\varvec{X}}}^{{\varvec{O}}}\end{array}\right],{\varvec{\Delta}}{{\varvec{X}}}^{{\varvec{r}}}=\left[\begin{array}{c}{\Delta X}_{1}^{r}\\ \vdots \\ {\Delta X}_{n}^{r}\end{array}\right]$$

$\Delta {X}_{i}^{r}$: Induced production of *i*-goods in *r*-region.

Induced Value-added vector

$${\varvec{\Delta}}{\varvec{V}}=\widehat{{\varvec{\nu}}}{\varvec{\gamma}}{\varvec{\Delta}}{\varvec{X}}=\left[\begin{array}{c}{{\varvec{\Delta}}{\varvec{V}}}^{{\varvec{C}}}\\ {{\varvec{\Delta}}{\varvec{V}}}^{{\varvec{N}}}\\ {{\varvec{\Delta}}{\varvec{V}}}^{{\varvec{S}}}\\ {{\varvec{\Delta}}{\varvec{V}}}^{{\varvec{O}}}\end{array}\right],\boldsymbol{ }{\varvec{\Delta}}{{\varvec{V}}}^{{\varvec{r}}}=\left[\begin{array}{c}{\Delta V}_{1}^{r}\\ \vdots \\ {\Delta V}_{n}^{r}\end{array}\right]$$

$\Delta {V}_{p}^{q}={\nu }_{p}^{q}\Delta {X}_{p}^{q}$: Induced value-added of *p*-goods in *q*-region.

${\varvec{\gamma}}$: Converter for consolidation of the industrial sector from 105 to 31.

Value-added ratio matrix

$$\varvec{\widehat{v}}=\left[ \begin{array} {llll}  \varvec{\widehat{v}}^{\varvec{K}} & \boldsymbol{0} & \boldsymbol{0} & \boldsymbol{0}  \\   \boldsymbol{0} & \varvec{\widehat{v}}^{\varvec{K}} & \boldsymbol{0} & \boldsymbol{0}  \\   \boldsymbol{0} & \boldsymbol{0} & \varvec{\widehat{v}}^{\varvec{K}} & \boldsymbol{0}  \\   \boldsymbol{0} & \boldsymbol{0} & \boldsymbol{0} & \varvec{\widehat{v}}^{\varvec{K}}  \\ \end{array} \right], \, \boldsymbol{ }{\widehat{{\varvec{\nu}}}}^{{\varvec{q}}}=\left[\begin{array}{ccc}{\nu }_{1}^{q}& \dots & 0\\ \vdots & \ddots & \vdots \\ 0& \dots & {\nu }_{n}^{q}\end{array}\right]$$

${\nu }_{p}^{q}=\frac{{GDP}_{p}^{q}}{{GDO}_{p}^{q}}$: value-added ratio of *p*-goods in *q*-region.

${\mathrm{GDP}}_{p}^{q}$: Gross domestic product of *p*-goods in *q*-region.

${\mathrm{GDO}}_{p}^{q}$: Gross domestic output of *p*-goods in *q*-region.

Superscript $q$ can be $K, J$ and represent Kumamoto Prefecture and Japan, respectively. Subscript $p$ represent industrial sector and can be from 1 to 31. This is because GDP and GDO can only be obtained from the prefecture and Japan and industrial classification of that is 31. Both value-added ratios are for fiscal year 2016. In addition, this analysis replace ${\varvec{\Delta}}{\varvec{F}},{\varvec{\Delta}}{\varvec{X}},{\varvec{\Delta}}{\varvec{V}}$ with the change of monthly expenditure ${\varvec{\Delta}}{\varvec{F}_{m}^{k\cdot l}}$, the induced production ${\varvec{\Delta}}{\varvec{X}_{m}^{k\cdot l}}$, and the induced value-added ${\varvec{\Delta}}{\varvec{V}_{m}^{k\cdot l}}$, respectively. Both vectors are equal in structure.

### Verifications of models and comparisons with actual values

Tables 7 and 8 shows the induced value-added coverage to the GDP for each region in FY2015-2016. The coverages indicate the extent to which value-added induced by targeted expenditure items covers actual values.

**Table 7:** Annual cumulative induced value-added coverage to GPDP

No.	Industries	(billion yen)					
		GPDP (production)		Induced value added		Coverage (%)	
		FY2015	FY2016	FY2015	FY2016	FY2015	FY2016
01	Agriculture	167	176	53	54	32	31
02	Forestry	13	13	1	1	10	12
03	Fishing	17	21	14	15	84	72
04	Mining	4	4	2	2	42	52
05	Food products and beverages	165	136	49	45	30	33
06	Textile products	17	15	2	2	10	12
07	Pulp, paper and paper products	35	38	3	3	10	9
08	Chemicals	101	83	20	21	19	25
09	Petroleum and coal products	4	5	2	3	61	54
10	Non-metallic mineral products	27	25	17	19	64	78
11	Basic metal	27	27	1	1	4	4
12	Fabricated metal products	62	74	10	13	17	17
13	General-purpose, production and business oriented machinery	142	157	7	6	5	4
14	Electronic components and devices	196	301	3	3	2	1
15	Electrical machinery, equipment and supplies	64	67	8	9	12	13
16	Information and communication electronics equipment	6	6	1	1	20	22
17	Transport equipment	93	89	3	4	3	5
18	Other manufacturing	124	125	23	22	18	18
19	Electricity, gas and water supply and waste management service	167	179	100	103	60	58
20	Construction	281	341	327	391	116	115
21	Wholesale and retail trade	579	595	337	335	58	56
22	Transport and postal services	268	274	248	240	92	88
23	Accommodation and food service activities	170	189	127	135	75	72
24	Information and communications	193	197	164	160	85	81
25	Finance and insurance	183	198	154	171	84	86
26	Real estate	607	605	602	557	99	92
27	Professional, scientific and technical activities	351	368	362	385	103	104
28	Public administration	393	410	4	4	1	1
29	Education	274	273	55	54	20	20
30	Human health and social work activities	634	643	43	43	7	7
31	Other service activities	289	281	166	149	58	53
32	Total	5653	5913	2910	2954	51	50

Consolidated industrial sectors from 105 to 31. The induced value-added was calculating using analytical model (7) and (8) with the total of the monthly expenditure each fiscal year as a given. The value-added ratio is applied for each fiscal year based on the National Accounts and Kumamoto Prefectural Accounts. The induced value-added is sum of all regions in the prefecture

**Table 8:** Annual cumulative induced value-added coverage to GRDP

No.	Industries	(%)					
		Kumamoto City		Northern Prefecture		Southern Prefecture	
		FY2015	FY2016	FY2015	FY2016	FY2015	FY2016
01	Agriculture	33	32	31	30	32	32
02	Forestry	8	9	11	12	9	11
03	Fishing	94	62	109	82	70	65
04	Mining and manufacturing	16	15	13	12	20	20
05	Electricity, gas and water supply and waste management service	46	43	57	50	71	74
06	Construction	113	107	125	115	94	121
07	Wholesale and retail trade	58	57	61	57	53	53
08	Transport and postal services	111	103	93	88	67	66
09	Accommodation and food service activities	63	65	91	80	73	72
10	Information and communications	125	121	36	33	32	29
11	Finance and insurance	89	88	74	82	77	86
12	Real estate	93	84	100	96	117	108
13	Professional, scientific and technical activities	65	67	203	188	119	147
14	Public administration	1	1	2	1	1	1
15	Education	29	28	14	14	8	8
16	Human health and social work activities	6	6	7	7	8	8
17	Other service activities	51	47	66	61	59	56
18	Total	55	53	48	46	50	52

Consolidated industrial sectors from 105 to 17 via 31. The induced value-added was calculating using analytical model (7) and (8) with the total of the monthly expenditure each fiscal year as a given. The value-added ratio is applied for each fiscal year based on the National Accounts and Kumamoto Prefectural Accounts

Although the targeted expenditure items accounted for about 80% of the GPDP (expenditure), Table 7 shows that their induced value-added covered only about 50% of the GPDP (production). By industry, the coverage was high for several services and fishing, and moderate for agriculture, food products, and wholesale and retail trade. In particular, the coverage in several manufacturing and related to public service sectors were noticeably poor. This suggests that the coverage is lower for industries with higher export rates or amount of government consumption. In addition, estimates for construction and professional activities exceeded actual values, while the forestry sector, which is not captured by the FIES, has a low coverage.

According to Table 8, the induced value-added by region also accounted for only about 50% of the GRDP (production) in each region. These areas are also in common to low coverage in industries related to exports and government consumption, and overrated construction. On the other hand, in northern and southern prefecture, the ratings of such as professional activities and information and communications were reversed comparing to the city, and they are each overrated.

If the analysis is limited to the targeted items, the estimated results for the prefecture can be said to capture the actual values to some extent. However, the weight of spending on exports and government consumption in induced value-added is too large to ignore. Moreover, the allocation of expenditures by region and industry confirmed a bias toward some sectors.

## Abbreviations

IO: Input–output table
MRIO: Multi–reginal input–output table
RDEI: Regional Domestic Expenditure Index
GDO: Gross domestic output
GDP: Gross domestic product
GPDP: Gross prefectural domestic product
GRDP: Gross regional domestic product
FIES: Family Income and Expenditure Survey
BSS: Building Starts Statistics
GRDFCF: Gross regional domestic fixed capital formation
MTCR: Monthly tourism consumption by region

## Footnotes

1 See the Appendix Table 3 for a detailed breakdown of the municipalities in each region.

2 “Information on the 2016 Kumamoto Earthquake,” Japan Meteorological Agency website. https://www.jma.go.jp/jma/menu/h28_kumamoto_jishin_menu.html. Accessed 6 August 2022.

3 “Damage from the Kumamoto Earthquake [Report 326],” Kumamoto prefecture, 2022. https://www.pref.kumamoto.jp/soshiki/4/51503.html. Accessed 6 August 2022.

4 Kumamoto Prefecture estimate: Kumamoto Prefecture, Agriculture, Forestry and Fisheries Department (2018). Cabinet Office estimate: Tsutsumi et al. (2016).

5 Welcome to Kumamoto, Tourism Promotion Plan (2017–2019), Kumamoto prefecture, 2018.

6 Verification Report on the Response to the Kumamoto Earthquake, the Department of Education, Kumamoto prefecture, 2018. https://www.pref.kumamoto.jp/site/kyouiku/9189.html. Accessed 3 May 2022.

7 Changes in occupancy of emergency temporary housing (As of March 31, 2022), Kumamoto prefecture, 2022. https://www.pref.kumamoto.jp/soshiki/27/132538.html. Accessed 3 May 2022.

8 Family Income and Expenditure Survey (April-March FY2016: Kumamoto City, households with two or more members), Statistical Bureau, Ministry of Internal Affairs and Communications.

9 Building Starts Statistics (monthly and annually FY2015–2018: Kumamoto prefecture), Ministry of Land, Infrastructure, Transport and Tourism. The housing types referred to here are "residential-only," "semi-residential," and "combined residential/industrial" housing on the BSS.

10 Subsidy system for travel plans with discounts to support tourism in Kyushu. The government has allocated 18 billion yen from the “Kumamoto Earthquake Recovery Reserve Fund” to each prefecture in Kyushu. The prefectures will request tourism-related companies to sell discounted products (trips with accommodations, one-day tours) and subsidize the amount of the discount.

11 In part of the supply constraint model, the struck areas are exogenized, so it is not possible to measure the ripple effects that extend to them.

12 Based on Chenery (1954), Isard (1951), and Moses (1955). See Takeda (2020) for details on how to prepare each intra- and multi-regional IO table.

13 According to the Guidelines for Prefectural Accounts Estimation Methodology of the Cabinet Office, consumption of households consists of expenditures by residents. Kumamoto Prefecture is considered to follow this rule, and tourism consumption is treated independently. However, since Kumamoto tourism statistics include in-prefecture tourists, it partially overlaps with consumption of households.

14 In fact, recovery and reconstruction is a long-term process. After April 2017, many people continued to be forced to live in temporary housing and private and public investment for lives back in order has expanded or remained at the high levels. However, Kumamoto Prefecture has been hit by a series of disasters, and various statistics do not exclude factors other than the earthquake. In other words, since it is difficult to isolate the earthquake alone from those factors, we focus on a short period of time when the impact of the earthquake is considered significant.

15 In the regional economy, the share of inter-regional balance of payments is high, and government consumption has been increasing rapidly due to the response to the earthquake, both of which are factors that cannot be ignored. However, since it is difficult to obtain monthly data, they are not included. The same is true for the other items.

16 Kumamoto city, households with two or more members, seasonally adjusted.

17 RAS method is based on Stone (1961).

18 Data for 2017 were not available, so estimates were made by linear interpolation from data for 2016 and 2018.

19 In the northern and southern areas, households and tourists within the prefecture are confused because of the different regional classifications in the Kumamoto Tourism Statistics and Kumamoto MRIO tables. Since it was difficult to adjust these figures, they were applied as is. Thus, for these overlaps, consumption in the northern and southern regions may have been overestimated.

20 Although the input coefficients and trade structures may change due to the earthquake, this paper does not take this into account due to data limitations. In this regard, an examination of the Great East Japan Earthquake by Kunimitsu and Ueda (2018) points out that the cost structure (input coefficients) is stable and the impact of disasters on the Leontief model is relatively small.

21 Consumption of households and tourism is converted by the distribution margin rate of the Input–Output table for Japan in 2015 to producer prices.

22 The adjusted self-sufficiency rate is applied for the calculation of tourism consumption.

23 The value-added ratio is calculated as the ratio of gross domestic product (GDP) to gross domestic output (GDO) by using the National Accounts and Kumamoto Prefectural Accounts for 2015 and 2016.

24 Since not all expenditure items are covered and the timing of the achievement of the ripple effects cannot be accurately measured. Thus, the induced value-added in this study do not match or exceed the GPDP as a rule.

25 Only in the southern prefecture is the net increase in value-added higher than growth of the GRDP, and it is much higher than in other regions. This suggests that there is a problem with the regional distribution of expenditures.

26 Please refer to the Appendix (“Verifications of models and comparisons with actual values” section).
